# Biomimetic Leadership for 21st Century Companies

**DOI:** 10.3390/biomimetics6030047

**Published:** 2021-07-14

**Authors:** Edita Olaizola, Rafael Morales-Sánchez, Marcos Eguiguren Huerta

**Affiliations:** 1 People Plus! Profit, 08195 Barcelona, Spain; 2 Department of Management and Marketing, Universidad Pablo de Olavide, Carretera de Utrera, km. 1, 41013 Seville, Spain; rmorsan@upo.es; 3 Director Chair in Sustainable Finance, UPF Barcelona School of Management, 08008 Barcelona, Spain; marcos.eguiguren@bsm.upf.edu

**Keywords:** organizational biomimicry, biomimetic leadership, leadership

## Abstract

Biomimicry is a scientific discipline that aims to model the behavior or properties of biological systems so as to adapt them to other scientific areas. Recently, this approach has been adopted in order to develop an organizational model called “Organizational Biomimicry”. It proposes a systemic approach, a worldview that places the organization and the people related to it as an integral part of nature, and an R&D system based on continuous learning from nature. The effective management of this business model depends on leaders who can make dynamic decisions, generate commitment to the views of the company, define specific goals, actively learn on multiple levels and tackle conflicts. This type of leadership may actually be being exercised in business practice; however, no leadership style inspired by biomimicry has been theorized to date. Thus, the aim of this research was to present a biomimetic leadership model that considers nature as a model, measure and mentor. To this end, we proposed, firstly, a definition of a biomimetic leader from the point of view of the characteristics of biomimetic organizations. Then, we determined the characteristics of this leadership type. Secondly, we conducted a review of the main leadership styles analyzed in the recent literature about management; then, for each leadership type, we extracted the characteristics that will adapt to the biomimetic leadership model. From this process, we obtained the traits of a biomimetic leader. This characterization (definition plus characteristics) was subjected to an expert panel, which determined its validity.

## 1. Introduction

Biomimicry, or biomimesis—from the Greek bios (life) and mimesis (imitation)—is a scientific discipline that aims to model the behavior or properties of biological systems to adapt them to other scientific areas. In the field of new material development, this approach has garnered extraordinary scientific achievements [1].

Biomimicry, as proposed by Benyus [2] and Pauli [3], has previously been successfully addressed in the fields of engineering, chemistry, robotics and architecture, among others [4,5,6,7,8,9,10].

It has also been applied to economic models [11] and organizational models [12], and as a system for the design and manufacture of products [13]. In the specific field of organizations, Celep, Tunç and Düren [14] have pointed out some aspects of management that can benefit from the biomimetic approach, such as corporate leadership, innovation, strategy and structure. However, until the recent work by Olaizola, Morales-Sánchez and Eguiguren-Huerta [15], the implications of this management philosophy for organizations had not been analyzed. Olaizola et al. [15] have characterized the so-called “Organizational Biomimicry”, adapting to organizations the 10 key points of the mature systems presented by Benyus [2]. According to Benyus [2], mature ecosystems consist of different beings that pursue common purposes: maintaining their presence in a certain place, making the most of the available resources, and prevailing in the long term. In a mature ecosystem, organisms [2]: (1) use waste as a resource; (2) diversify and cooperate to fully use the habitat; (3) gather and use energy efficiently; (4) optimize rather than maximize; (5) use materials sparingly; (6) do not foul their nets; (7) do not draw down resources; (8) remain in balance with the biosphere; (9) run on information; and (10) shop locally. From this analysis of mature systems, “it can be inferred that biomimetic organizations present light and even fluid structures, multidisciplinary teams working on projects, value management, ecosystem vision, investment in R&D focused on nature, and distributive and ethical leadership” [15] (p. 12).

Organizational biomimicry proposes a systemic approach, a worldview that places the organization and the people related to it as an integral part of nature, and an R&D system based on continuous learning from nature.

Olaizola et al. [15] (p. 17) conclude that the biomimetic management model contributes to the organization in the following ways: “(1) stimulation of creativity, pride of belonging, commitment and the well-being of people who interact with it directly or indirectly; (2) optimal use of all resources; (3) understanding and imitation of the vital mechanisms of the Earth that need to be respected and protected; (4) continuous improvement in the definition and implementation of strategies and policies; (5) innovative and disruptive products or services; (6) active involvement of customers and suppliers; (7) triple bottom results sustained over time; (8) complicity with society; (9) conservation of the planet for future generations (human and non-human); and (10) welfare in the organization subsystem and in the other subsystems with which it interacts directly or indirectly”.

The effective management of a complex business model depends on leaders who can make dynamic decisions, generate commitment to the views of the company, define specific goals, actively learn on multiple levels and tackle conflicts [9]. Therefore, according to Olaizola et al. [15] (p. 13): “If we want a biomimetic organization to count on its internal ecosystem with people endowed with the aforementioned characteristics, it is necessary to provide the means for relying on leaders capable not only of having them, but of self-evaluating and committing themselves to continuous improvement. Leaders who are capable of directing the common project that is an organization”.

While this type of leadership may actually have been exercised in business practice, to the best of our knowledge, no leadership style inspired by biomimicry has been theorized to date. Thus, the aim of this study was to present a biomimetic leadership that considers nature as a model, measure and mentor. To this end, we proposed, initially, a definition of a biomimetic leader derived from the characteristics of biomimetic organizations. Then, we determined the characteristics of this leadership type. For this purpose, we conducted a review of the main leadership styles as analyzed in the recent literature about management; then, for each leadership type, we extracted the characteristics that will adapt to the biomimetic leadership model. From this process, we obtained the traits of the biomimetic leader. This characterization (definition plus characteristics) was subjected to an expert panel, which determined its validity.

Our work offers a double contribution to the literature. Firstly, from the biomimetic perspective, this is the first time that such an approach has been applied to the management of people, which shows its potential as a holistic discipline, capable of contributing to science beyond bioinspired mechanical systems. Secondly, from the perspective of management, the biomimetic leadership model presented here is a view that surpasses the existing anthropocentric systems in the literature and in the practice of business management. Using a multidisciplinary and integrating perspective, biomimetic leadership is founded on the advances of perspectives such as biomimicry, psychology, pedagogy, management, and even esthetics and engineering, to propose a novel view of leadership inspired by nature. 

This multidisciplinary approach has proved useful in previous studies; for instance, Pérez Matos and Setién Quesada [16] (p. 15) stated that “the complexity of current reality forces the scientific study of society as a whole and of the individual person in his/her values and rules. This leads to a new stance in the treatment of social sciences that, from inter- and trans-disciplinary approaches, allows creating different epistemological structures in the sciences that are inherent to the study of societies”. In this sense, Rahwan et al. [17] suggest that we must study artificial intelligence in the same way that we study wild animals, with the interaction of computation scientists, physicists, climate experts, architects, philosophers, psychologists and sociologists. 

In specific fields, Jarke et al. [18] used it in the management of scenarios, using an inter-disciplinary approach that comprises strategic management, person–machine interaction and system engineering; Dietz [19] studied the influence of the community on the individual social or economic results from sociology, economy and geography; Antonucci et al. [20] studied the dominant influence of relationships on health and well-being, based on different disciplines; Hiwasaki and Arico [21] integrated social sciences with eco-hydrology.

This paper is structured as follows. In the next section, we present the definition of biomimetic leadership from the viewpoint of the characteristics of biomimetic organizations. Next, we describe the research methodology. The subsequent sections present the characteristics of the biomimetic leadership model, extracted from the review of the main leadership styles in the recent literature about management and validated by an expert panel. Finally, discussion and conclusions are drawn.

## 2. Definition of Biomimetic Leadership

The literature does not provide a model of the biomimetic leader. Thus, in order to approach it, its characterization must begin with an ethical leader that recognizes him/herself as part of the natural system with which he/she interacts—and learns—to obtain mutual benefits. The biomimetic leader is an ethical leader endowed with a worldview that considers nature as a model, measure and mentor, and such a continuous learning process generates observable and measurable behaviors that benefit the organization. According to these ideas, a biomimetic leader defines strategic goals transversally and consensually within the different subsystems of the organization, incorporates the attainment of goals into the considerations assigned to the participants, and shows and favors behaviors that promote the co-responsibility of all participants.

From the characterization of biomimetic organizations proposed by Olaizola et al. [15], we can present a definition of the biomimetic leader. Thus, we define the biomimetic leader as a person committed to his/her own personal and professional growth, emotionally involved in the organization for which he/she works, and willing to accompany it in its process of development, in harmony with the environment. His/her source of inspiration and learning is nature, and he/she strongly believes that he/she and the organization are nature, are part of nature, belong to one of the ecosystems of nature and are responsible for the health and well-being of nature. 

The biomimetic leader must have a more complex profile with respect to the traditional concept of an ideal leader, regardless of the leadership model. Therefore, to characterize biomimetic leadership, we first reviewed the main theories about leadership in order to extract the most effective traits and behaviors of the leadership models explored in the literature on business organization. Secondly, we applied the philosophy of biomimicry to characterize a leader endowed with a worldview that considers nature as a model, measure, and mentor. Lastly, we subjected our proposal to an expert panel, who evaluated it and made contributions for its improvement.

## 3. Methodology

Management, as a social science, requires research methodologies that are capable of collecting all the complexity of business phenomena and analyzing them. We adopted a grounded theory approach to develop qualitative research. The “grounded theory” approach has become a popular choice of methodology among social researchers in recent times, and it is an approach dedicated to generating theories. In this sense, it contrasts with approaches concerned with testing theories, and is different from research where the main purpose is to provide descriptive accounts of the subject matter [22]. To attain the objective of our research, we developed a two-step process: first, we analyzed the current literature about leadership in management to extract the characteristics that adapt to the biomimetic leadership model. Second, this characterization (definition plus characteristics) was subjected to an expert panel, which determined its validity.

## 4. Review of the Leadership Models

This section briefly presents the main contributions that can be obtained from the leadership styles analyzed in the recent literature. Given that there are a large number of leadership styles, we selected for our research those that meet two criteria: (1) those leadership styles that have proved more effective for organizations; and (2) those that appear to be related to the biomimetic approach. According to the first criterion, the selected leadership styles are transformational, authentic and ethical leadership [23]. Based on the second criterion, we chose the following leadership styles: servant, sustainable, creative, distributed, spiritual, holistic, innovative and regenerative leadership. The aim of this study was not to dissect the leadership styles; thus, instead of attempting to thoroughly analyze each of them in our literature review, we present them briefly here and highlight the most important aspects of each style, as well as their possible association with biomimesis or organizational biomimicry. 

The analysis of these leadership models or styles must serve as a basis to determine the traits of a leader who considers nature as a model, measure, and mentor, and who aims to behave like the organisms of a mature system, that is, a biomimetic leader.

### 4.1. Transformational Leadership

Transformational leaders are those who inspire their followers to transcend their personal interests for the good of the organization, while paying attention to their concerns and needs, and thus having an extraordinary effect on them. According to Avolio and Yammarino [24], the transformational leader has values and beliefs as well as a sense of mission and purpose (his/her duty). He/she has a moral and ethical orientation and inspires and motivates. He/she applies the Pygmalion effect and creativity for innovation and problem-solving and provides individualized socioemotional support to each of his/her followers. 

Bass [25] suggested four key aspects of transformational leadership: idealized influence and charisma (behavioral models with high ethics); inspirational motivation (great team spirit and a shared view); intellectual stimulation (emphasizing problem-solving and creativity); and individualized consideration (a supportive climate and the use of delegation). Other authors, such as Avolio and Locke [26], Kirkpatick and Locke [27], van Dierendonck and Patterson [28], and Bass and Steidlmeier [29], also include moral concepts in this type of leadership (e.g., virtue, charisma and commitment to the common good). 

The way in which transformational leadership manages individualized consideration and provides individualized socioemotional support to every follower is in line with the second requirement of mature systems proposed by Benyus [2] (to diversify and cooperate to fully explore the habitat), and also with the tenth requirement (shop locally). Moreover, intellectual stimulation and the promotion of creativity for innovation and problem-solving is a necessary skill for a leader who aims to have nature as a model and mentor and, thereby, learn from it. Lastly, the altruism required in transformational leadership is linked to the generosity demanded from people in the characterization of biomimetic organizations.

### 4.2. Authentic Leadership

The study of authentic leadership is also gaining great importance. In this leadership style, the leader believes in the values and beliefs of the group, which makes the latter have faith in the former. According to Walumbwa et al. [30], authentic leadership has four axes: self-awareness (understanding one’s strengths and weaknesses, and the influence of the development of one’s behavior), transparency in relationships (sharing information openly and expressing honest thoughts and feelings), the development of a moral perspective (setting high ethical standards for behavior) and balanced processing (analyzing information objectively and asking for the opinions of the followers before making a decision). 

Authentic leaders generate confidence, optimism, resilience and moral strength [31], and offer greater social identification with the group and organizational principles [32]. Furthermore, they behave in such a way that they show self-transcendence values, such as honesty, loyalty and equality [31]. 

Moriano et al. [33] stated that authentic leaders are individuals who are highly aware of their values and beliefs, how they behave, and how they are perceived by others. On their part, Shamir and Eilam [34] pointed out that the behaviors of the authentic leader are based on high levels of self-knowledge and a concept of self, coherence and harmony between the person and the role, and that such behaviors make up a biography that attracts his/her followers. 

It is easy to extract traits of authentic leadership that are in line with the biomimetic approach of organizations and mature systems. Specifically, the trait of transparency in relationships (sharing information openly and expressing honest thoughts and feelings) as proposed by the authentic leader corresponds to the ninth point of mature systems: they are governed by information. For an authentic leader, authenticity in communication becomes honesty (saying what one thinks, doing what one says). The resilience generated by authentic leaders is a way of developing the eighth point of mature systems: remaining in balance with the biosphere through diversity, redundancy and decentralization.

### 4.3. Ethical Leadership

These approaches can be enriched by adding the importance of ethics in leadership. As was indicated by Melé [35], ethics must be at the core of the management of organizations, and thus in their leaders, in order to make decisions according to good morality, mold the management style, strengthen their virtues, implement good practices in the organization and exercise an adequate leadership model. 

Ethical leadership is defined as “the demonstration of normatively appropriate conduct through personal actions and interpersonal relationships, and the promotion of such conduct to followers through two-way communication, reinforcement, and decision-making” [36] (p. 120). The ethical leader, in addition to showing behaviors in line with the rules, promotes, in his/her inter-personal relationships, the incorporation of such behaviors by the members of his/her team, and he/she does that basically in two ways—strengthening adequate behaviors and discouraging behaviors that are not in line with the rules. According to Marsh [37], the ethical leader must have four traits: authenticity (personal integrity, self-knowledge and the capacity to manage his/her own life); mindfulness (the capacity to observe, the capacity to take time to think, systemic thinking, rational process); sustainment (hope and a holistic approach in the workplace and in life); and commitment (acceptance of diversity, cultivating relationships, and agreeing to make risky decisions).

Ethical leadership has shown its positive effects on pro-social behaviors [38] and has been positively related to psychological well-being and job satisfaction among workers [39,40]. In the same line, Engelbrecht et al. [41] emphasize the key role of ethical leaders in the creation of an ethical and trusting work climate that favors the participation of the employees. Other authors have positively associated ethical leadership with organizational commitment, emotional commitment, normative commitment, organizational citizenship behavior, work performance, work commitment and organizational identification [42]. On their part, Kopelman et al. [43] reported that ethical leadership reduces the immoral behaviors of the subordinates. 

For our characterization of biomimetic leadership, we highlight the following traits of ethical leadership: integrity, systemic thinking, a holistic approach and commitment.

### 4.4. Servant Leadership

Servant leadership is focused on the service to others [44,45] as it is the highest moral dimension [46], and it recognizes that the duty of organizations is to create people who can build a more humane future [47] and are capable of solving the challenges of 21st-century society. 

According to Greenleaf [44], the servant leader: is a good listener; is empathetic; is a healer; becomes aware; is persuasive; can conceptualize, i.e., consolidate his/her capacities “to dream great dreams”; exercises foresight; knows how to administer; is strongly committed to the growth of people around him/her; and promotes the construction of a community among those who work within a certain organization. 

There is a positive relationship between the practice of the servant leader and the organizational commitment [48,49] or civic behavior of the followers [50,51]. Employees can notice that, in order to maintain a high service level, the organization/leader greatly appreciates behaviors that go beyond the formal requirements, i.e., a greater civic behavior [52]. Moreover, the servant leader can improve the social communication and integration of the organization/group [53]. Co-responsibility also indicates that the priority of servant leaders is to satisfy the needs of the followers and society before attaining the goals of the organization [47,54,55].

Servant leadership is linked to biomimicry through the capacity to commit to the growth of the people around the leader and through the promotion of community-building, which are requirements of mature systems. Furthermore, due to its special orientation to serve the organization and others, the traits of honesty, commitment, civic behavior, social communication and integration, co-responsibility, integrity and humility are especially important for a biomimetic leader.

### 4.5. Sustainable Leadership

Gerard et al. [56] state that sustainable leadership requires: (1) a long-term perspective for decision-making; (2) promoting systematic innovation aimed at increasing value for the client; (3) developing a qualified, loyal and strongly committed human team; and (4) providing quality products, services and solutions. The practices of sustainable leadership allow generating a quick and solid response that is competitive and attractive to the participants [57,58]. 

In their definition of the concept, Avery and Bergsteiner [59] claim that sustainability is the fundamental principle of leadership. The existing definitions of sustainable leadership highlight a series of characteristics and traits that sustain the concept. Briefly listed, the key ideas are as follows:

- Depth—sustainable leadership matters;
- Duration—sustainable leadership lasts;
- Amplitude—sustainable leadership expands;
- Justice—sustainable leadership does not harm the environment and actively improves it;
- Diversity—sustainable leadership promotes diversity and cohesion;
- Inventiveness—sustainable leadership develops and does not deplete materials in human resources;
- Conservation—sustainable leadership, honors and teachings are the best of the past to create an even better future.

In addition to incorporating corporate social responsibility (CSR), Kantabutra and Avery [58] compared organizations with nature. They also insist on social responsibility toward the environment, in terms of respecting it along with the people and the community.

Casserley and Megginson [60] tackled sustainable leadership, stating that its meaning or purpose is based on something deeper and more permanent than merely achieving professional goals, and that it goes beyond the narrow interests of the leader. Such leaders also have a very well-developed reflective capacity, making sense of things at an emotional, intuitive and intellectual level, and responding in a more visceral manner; thus, they are capable of reconsidering, self-criticizing, and creatively adapting to changes in their environment. 

Regarding all of the abovementioned models, sustainable leadership contributes to the previous leadership styles with a special emphasis on CSR, the relationship between the organization and its environment, and the special importance of diversity.

### 4.6. Creative Leadership

Sternberg et al. [61] concluded that there are three general types of creative leadership: (1) the leadership that accepts existing ways of doing things; (2) the leadership that challenges the existing ways of doing things; and (3) the leadership that synthesizes different existing ways of doing things. They also stated that this leadership style can be applied through the following procedure: (1) replicate, (2) redefine, (3) increase, (4) advance, (5) redirect, (6) rebuild, (7) reinitiate, and (8) synthesize/fuse. 

Palus and Horth [62] considered that the main competencies of creative leadership are interrelated, although they are different from each other: attention, personalization, the creation of images, fair play, collaborative inquiry and craftsmanship. They also proposed that leadership processes are fundamentally artistic, identifying artistic creation as the representation of specific and powerful ways of perceiving, building, mobilizing and participating in evolving realities. 

Basadur [63] pointed out that leaders must first help others to perform the basic skills of creative thinking in order to overcome deficiencies. These skills not only help to solve obvious problems (problem-solving) but they also help people to follow a synchronized process toward innovative thinking in order to find and define new problems, with the aim of solving and implementing the new solutions.

Leaders must help others to follow this process, not only individually but also with other individuals or within groups or teams. These basic thinking skills include the ability to defer judgment, maintain an open mind and think divergently. DiLiello and Houghton [64] insist that creative ideas can be used to solve problems, improve processes and develop new services and/or products. These authors highlight that individual creativity is essential for organizational innovation, and they point out personal traits such as perseverance, curiosity, an interest in complexity, a preference for autonomy and high energy. 

Regarding biomimicry, we consider that a leader who learns from nature and considers it as a mentor must resort to creativity in order to bring natural processes and models to the organizational structures and processes. Therefore, creativity must be a defining trait of biomimetic leadership.

### 4.7. Distributed Leadership

The literature about distributed leadership generally refers to the school environment (e.g., the professional learning communities described by DeMatthews, [65]), although, since schools are organizations, we can extract useful teachings for organizations in other environments, including companies. 

Distributed leadership is essentially scattered, that is, it is not focused on one individual or on a single level. It is fostered by a reality in which more than one person can be a manager, regardless of the differences established by the management of the organization. This favors equal opportunities in order that all the members of an institution can lead and make decisions at a certain moment.

From a holistic perspective, leadership is viewed as an arranged action, where it is a phenomenon that comprises the practice of delegation, exchange, collaboration, dispersion and the democratization of leadership. Longo [66] explained that an organization that applies distributed leadership makes people act as leaders while they fulfill their habitual duties. 

Distributed leadership acquires interest due to the background tendencies of current knowledge-based organizations, societies and economies, in more demanding environments, with an increase of internal diversity or flatter hierarchical structures. In this regard, Longo [66] defined distributed leadership as: an attribute of organizations; a resource that should be aimed at orienting, creating/maintaining motivation and managing changes, with a mainly contingent character; and as a moral authority and an exercise of influence to convince and solve conflicts, with a preferably delegating style (although transferring leadership should not be mistaken for transferring work).

The underlying notion of distributed leadership, a scattered leadership shared according to the activity and profile of each member of the team, is related to the eighth characteristic of mature systems proposed by Benyus [2], i.e., the balance of the biosphere, specifically through diversity, redundancy and decentralization.

### 4.8. Spiritual Leadership

Spiritual leadership is focused on integrity, honesty, compassion, respect and humility [67]. All these values show the followers that the spiritual leader is a person they can trust [68]. In this sense, Morales-Sánchez and Cabello-Medina [69] propose that a good leader must have at least one of the following competencies: transcendence, spirituality, religiosity, or emotional sense. 

Sharma [70] emphasized that spiritual leaders manage to create a clear design or plan of how their companies will be in the following years. Blanch et al. [71] argue that spiritual leaders develop an inspirational view and mission that promote the development of the spirit of cooperation, mutual support and commitment to the effective functioning of the organization. 

In this sense, Benefiel [72] studied the importance of spirituality in the transformation process of organizations, concluding that spiritual leaders who are focused on their organizations (in the same way as spiritual teachers who are focused on people) can resort to the collective knowledge of the experience of others and compassionately accompany their organizations through their transformation. This demystifies the leadership process and allows the organization to realize that it is not alone but is instead accompanied by a set of people who have grown through their life experiences. 

The characterization of biomimetic organizations proposed by Olaizola et al. [15] insists on the importance of the ethics of the organization and of the people that constitute it. Moreover, these authors refer to the importance of reflecting on the mission of organizations and the coupling of higher systems to the organization. All these factors are clearly represented in spiritual leadership, among which we highlight respect, humility and life sense.

### 4.9. Holistic Leadership

Best [73] proposed that holistic leadership offers seven fundamental assumptions about the nature of effective leadership:

1. Successful outcomes result from an orientation toward development;
2. The healthiest and most productive development is performed collaboratively;
3. The leadership unit shapes the context of collaboration;
4. The core leadership unit is the individual, which makes every participant a leader within his or her own sphere of influence;
5. The intrinsic desire for meaningful purpose suggests that every individual wants to realize his or her best potential;
6. Holistically led collaboration requires that the participant’s right to self-determination be respected;
7. The exercise of self-determination in a way that realizes the individual’s best potential results from an iterative process that must be supported.

On their part, Korhonen et al. [74] stated that the orientation toward goals, interactions and the exchange of responsibilities (shared leadership) are key characteristics of school leadership. The characteristic effects of leadership that workers must show are participation, empowerment and commitment. 

Focusing on the school context, Niemi [75] claimed that schools require cooperation among professionals, which is a perspective centered on learning, networks within the school community and with external agents, and long-term development plans. In addition to internal collaboration, external collaboration networks are also important, which include the parents of the students, the local community organizations, the companies, and other national and international networks. 

Although there are currently few studies on holistic leadership, the stress they place on collaboration, network development, self-determination and, obviously, the holistic perspective, makes this a very interesting leadership style for the biomimetic approach, since these are essential characteristics of mature ecosystems [2].

### 4.10. Innovative Leadership

Ditkoff [76] highlighted the following behavioral traits of creative people: they usually question the status quo; they investigate new possibilities; they are self-motivated; they are concerned about the future; they see possibilities in the impossible; they take risks; they tend toward movement and interaction; they do not fear ridicule; they see hidden connections; they focus on challenges and problems; they are perceptive; they resist ambiguity and paradoxes; they learn constantly; they can reconcile intuition and analysis; they communicate effectively; they are not easily discouraged; and their individualism does not prevent them from working in a team if they are given space.

González-Romá [77] considered that, in the fulfillment of their duties, leaders can facilitate innovation by contributing to the development of certain group processes: clarifying the goals of the team and creating a shared view of the latter, stimulating participation in decision-making, periodically allocating a time for team reflection, managing conflicts cooperatively, and supporting the implementation of new ideas.

With respect to innovation in work teams, Bornay-Barrachina [78] distinguished between “associative” thinking and “bisociative” thinking. Associative thinking is limited to the search of solutions along already known paths, looking for associations between different pieces of knowledge and experiences that have been accumulated and which are frequently used. On the other hand, bisociative thinking relates scattered concepts. These behaviors consist, for instance, in searching for new approaches that are not required at the time or combining ideas from different research areas.

Biomimicry requires innovation and creativity to adapt what is learned from nature to different environments, processes and systems. As was explained earlier regarding creative leadership, these traits of the leader (creativity and innovation capacity) are essential in organizational systems that face continuous change, and which aim to consider nature as a teacher and be inspired by it.

### 4.11. Regenerative Leadership

Hutchins and Storm [79] (p. 84) point out that regenerative leadership is based on the multidisciplinary fields of the theory of complexity, cybernetics, evolutionary psychology, systems theory, holistic science and others: “For sure, we take learning from nature’s ways of communicating, evolving and collaborating that have been honed over billions of years of evolution, and we combine this with recent findings about energy flows in complex adaptive systems, detailed studies on adult development growth within organizations, feedback loops within system dynamics, and more. All this contributes to a rich picture of how we view the life-organization-as-living-system that thrives through messy human relationships nested within systems upon systems of life”. A good practical example of this is provided by Mangrich et al. [80], who explained how a physical university space is used as a pedagogical instrument for the resilience of ecosystems. Some characteristics of regenerative leaders are presence, coherence, patience, generosity and silence [79]. From regenerative leadership, organizational biomimicry can extract behaviors that tend toward the integration of different disciplines and the development of transformative ethics.

Table 1 shows the reviewed leadership models, a summary of their main characteristics and some of the published studies about them.

After reviewing the main leadership models that appear in the specialized literature on management, the next section presents the characterization of the biomimetic leader through the traits that compose this profile.

## 5. Results: Characterization of the Biomimetic Leader

To carry out this characterization, we used the information extracted from the reviewed leadership models. For each model, we extracted the leadership traits that fit the characteristics of biomimetic organizations or the mature ecosystems described by Benyus [2]. 

From the analysis of the leadership models, we extracted the following traits: creativity, ethics, flexibility, generosity, honesty, humility, loyalty/commitment, resilience and a holistic view. Table 2 shows the types of relationships established between the traits of the biomimetic leader and the 10 key elements of a mature system [2].

In addition to the traits found in the literature, we believe that the biomimetic leader must have two more traits: a sense of esthetics and a sense of humor. However, these two traits have no parallels in the model of mature systems developed by Benyus. This could be due to the fact that such a model mimics nature to design products and processes, that is, activities that can be approached from a “mechanic” perspective, since they respond to predetermined, predefined and pre-valued guidelines. Our proposition of biomimetic leadership goes one step further, as it integrates the whole of human complexity into the model, not from an anthropocentric viewpoint, as is usual, but from a new approach based on world view. 

The following section thoroughly explains why “sense of esthetics” and “sense of humor” were included in the traits of the biomimetic leader, despite not being among the main traits of the leadership models, and in spite of the absence of similarities among the key elements of mature systems.

### 5.1. Sense of Aesthetics

This is a desirable trait in a biomimetic leader, as it is related to:

(a) Leader growth: esthetic comprehension lies in the sensory capacities [92]; esthetic pleasure implies the exercise of intellectual capacities [93]; beauty is the gateway to the higher world [94]; and esthetic pleasure is knowledge, a “thinking pleasure” [95].
  According to Cingari [96], esthetics is a theoretical experience separated from science and morality that exists in every human activity. Similarly, it can help to expand viewpoints, since, as was pointed out by Cassirer [97], while science abbreviates and impoverishes reality in its abstract presentation, art specifies and intensifies it. Regarding art, Pugh and Girod [98] reported that interacting with art objects transforms people, since the objects provide moments of pleasure, expand our horizons and modify our ways of perceiving the world, leaving us irrevocably changed.
(b) Leader’s relationship with the organization: esthetic creations transform reality, as they are artistic and human novelties [99]. Esthetics is a fundamental part of the organization [100], and transformative organizations incorporate esthetic properties and use different esthetic symbols [101]. Furthermore, as was stated by Dewey [102], there is a strong relationship between esthetic experience and ordinary experience, thus every activity can be esthetic if it is fully conducted and is thereby strongly linked to continuous improvement.
(c) Leader’s relationship with nature: we need to restore nature’s esthetics to save the biosphere [103]. According to Miguel de Unamuno, “Only a few feelings can provide greater solace to man in his sorrow, more rest in his works, more tranquility in the midst of struggles for survival and more serenity of mind than the feeling of Nature. When one has it with some liveliness, gazing at the countryside is the best sedative for the diseases of the spirit. Inhaling the landscape is one of the greatest pleasures of life” (quoted in [104], translated from Spanish).
  Likewise, Tafalla [103] (p. 222) argued that “Nature, which is not our creation, lacks limits and frameworks, and not only allows us but encourages us to enter and discover it. The esthetic experience of nature is the experience of something that opens up to welcome us, surrounding us. We stop being mere distant spectators to find ourselves inside it, participating in it, discovering ourselves as inhabitants, as members of the natural world. A passer-by roaming a landscape becomes part of that landscape, and thus nature is the only artwork we can be a part of as physical individuals, in our corporality” (translated from Spanish).

### 5.2. Sense of Humor

A sense of humor is a desirable trait in a biomimetic leader, since it helps him/her in his/her process of continuous improvement in three different ways:

(a) Leader growth: Carbelo and Jáuregui [105] claimed that a sense of humor, as a personality trait, is one of the main strengths of human beings. In fact, humor implies self-improvement, acceptance and tenderness, and it is also an attitude of coherence [106], as it strengthens self-concept and self-esteem [107] and helps in the learning process, to such an extent that “humor and learning are naturally bound” [106] (p. 12).
  A sense of humor allows setting the identity and personality of people and groups, as it provides them with tools with which to build the reality of their common and shared lives. In this regard, Vázquez [108] commented that a sense of humor is the culmination of a proactive attitude to reach self-knowledge and achieve self-acceptance, which allows joking and laughing, even at ourselves.
  In fact, humor is a good ally to perceive the stimuli of the environment, and, in its self-asserting sense, is a mechanism of emotional regulation, as it allows people to laugh at the inconsistencies of life and keep a humorous perspective, even in the face of adversity [109]. As was indicated by Betés de Toro [110], those who usually practice humor continuously reflect on their life sense, developing and maintaining an attitude of acceptance. A sense of humor is also a manifestation of itself, as an act of maturity, according to Labarca Reverol [111].
(b) Leader’s relationship with the organization: Humor helps the leader to establish close relationships with the members of the team, learn from the people around him/her, improve his/her interpersonal relationships and trust others; it also favors the analysis of different situations from different perspectives to address all the nuances, and helps the team to build the reality of their common life [112]. Yam et al. [113] state that the leader’s sense of humor increases the work commitment of the followers: the employees feel safe and confident to be themselves, which in turn allows them to fully invest their personal energy in their job duties. In this respect, Beard [114] demonstrated that humor at work is an accelerator of productivity. According to Mann [115], humor is the antidote for boredom/boring tasks, and can be used to enrich society, since it is a great catalyzer of creativity, thinking and intelligent reflection. Even in nature, Panksepp [116] asserts that laughter is the best measure of social pleasure in animals.
(c) Leader’s relationship with nature: Applying humor to learning from nature helps the leader to visualize the old rules, thus facilitating a new view, becoming aware of his/her expressive behavior at an almost innocent level, according to Aladro Vico [117].
  Furthermore, a sense of humor can be a magnificent tool for the biomimetic leader to raise awareness in his/her group, organization, environment and all participants about the importance of knowing nature in order to love it, thus favoring more respectful behaviors toward it. According to Hollman [118] (p. 228), “the relationship between humoristic images and environmental issues provides interesting elements to understand the role of the visual culture in the construction of feelings and meanings about nature and its transformation” (translated from Spanish).

This analysis of leadership traits contemplates the enhancement of certain personality traits; however, not all traits are adequately tackled. Thus, at this point of the analysis, we aimed at completing our proposition by incorporating the contributions of transactional analysis (TA). According to Steiner [119], Steiner and Devós [120], and Casado Esquius [121], TA shows the benefits of working with three states of the “Self” that make up a mature personality. Harris [122] explained that there are three states in each person: it is as though in each person, there exists the same creature that he/she was at the age of three years. Secondly, one’s parents are also within each person, in the form of the cerebral recordings of authentic experiences of internal and external events, among which the most important ones took place during the first five years of life. The third state is called “the Adult state”, whereas the other two are called “the Parent and Child states”. These three states of the self are psychological realities.

Therefore, we can summarize that the “Parent state” of the self corresponds to the values and principles of a person, the “Adult state” is in charge of analysis in order to make decisions, and the “Child state” contributes with feelings and emotions. Therefore, our proposition requires the leader to have all his/her personal resources available; in other words, the “Child state” must have the relevance it deserves in life along with the “Parent” and “Adult” states. As is shown in Table 3, the traits of a sense of esthetics and a sense of humor contribute to enriching the “Child state” of the self, thereby achieving a balanced and rich person capable of managing all his/her resources.

The “Child state” of the self was first studied as “Emotional Intelligence”. Mayer et al. [123] measured emotional intelligence, which they defined as the capacity to perceive, value and express emotions accurately, generate feelings that facilitate thinking, understand emotions and emotional knowledge, and regulate emotions, promoting emotional and intellectual growth. On their part, Goñi Palacios and Fernández Zabala [124] considered that personal self-conception is composed of emotions, honesty, self-realization and autonomy, and that feelings are part of perception, according to the definition of Farlex [125]. Mann [115] concluded that emotions consist of 4 components: our knowledge, our feelings, our physiological reactions and our behaviors. 

Therefore, a mature person also uses the “Child state” of the self; as was explained by Harari [126], even an economist who has won the Nobel Prize makes a very small part of his/her decisions using a pencil, paper and a calculator. By this argument, 99% of our decisions (including the most important ones in life, related to partners, careers and habitats) are made by highly refined algorithms called feelings, emotions, and desires.

After incorporating the traits “sense of esthetics” and “sense of humor”, the biomimetic leader is characterized by 11 traits. Table 4 shows the traits of the biomimetic leader and the definition of each trait, based on the literature review.

### 5.3. Testing the Defined Profile: Expert Panel

After theoretically determining the concept and traits of the biomimetic leader, such characterization was subjected to a panel of experts to obtain validation based on their experience and knowledge. The aim of this expert panel was to produce a shared view from a group of people with extensive knowledge about a certain subject by aligning their opinions, which were gathered in the form of questionnaires. This method proved to be very useful for improving knowledge in complex, novel and future matters (forward planning). Since the existence of biomimetic leaders as such is unknown, the questionnaire was sent to 18 people who met two criteria: (1) having proven experience in managing work teams; and (2) having demonstrated good levels of ethics in the exercise of their profession. In Appendix A you can see the profile of the selected experts. The questionnaire designed for the experts began with our definition of a biomimetic leader, followed by a question about the traits that, in their opinion, were required in biomimetic leadership, on a scale of 1 to 5. 

Table 5 shows the results of this question classified in order from highest to lowest, based on the scores obtained.

In response to a suggestion from the expert panel, we considered it relevant to remove the trait “honesty”, as it is very similar to “ethics” and “loyalty/commitment” (ethics obtained a total score of 85, whereas loyalty /commitment and honesty obtained 80 points each).

We understand that the result obtained in the expert panel was positive, since all the traits were above the average value, despite the novelty of the proposition; moreover, positive comments were also obtained. It is worth highlighting, also positively, that the new traits provided by this investigation (i.e., sense of esthetics and sense of humor) were classified as “brave” and, nonetheless, obtained scores above the average value, although obviously slightly below those traits that we could consider to be “traditional”. It is important to take into account that people need a period of adaptation to change, as is the case with accepting “sense of esthetics” and “sense of humor” as relevant traits for a leader (biomimetic). Bridges [127] specified that a process of change from the personal perspective implies going through three states: (1) the end of a period (releasing known things, “letting go of” ideas, belongings, etc.); (2) a transition zone, a necessary bridge (the valid reference so far disappears, the focus is lost and anxiety appears); (3) the beginning of a new stage, first steps in a different reality that is not well-defined as yet. 

This capacity to accept change (preferably shortening the adaptation phase) is especially valuable in this period, in which the planet faces a climatic emergency; even large consultancy companies such as Mackinsey and Company (of great professional prestige, and clearly anthropocentric), in their report of January 2020, signed by Woetzel et al. [128], offer organizations new methodologies designed to help in the process of change, focusing on risk assessment in fields such as habitability, job opportunities, food systems, architectural risks, infrastructure and ecosystems.

## 6. Discussion and Conclusions

The aim of this study was to characterize the biomimetic leader, by reviewing the literature on leadership and gathering contributions from other disciplines like biomimicry and transactional analysis. The result of our investigation is a theoretical construct, “biomimetic leadership”, which is characterized by ten traits. Since such characterization is new, it was subjected to a panel of experts, who validated the main propositions of our study and helped to refine its content. Following the suggestion of the experts, one of the traits was removed or rather included within another trait. Specifically, the trait “honesty” disappeared due to its similarity with the trait “ethics”. The coexistence of these two similar traits could have resulted in methodological and content difficulties. 

When describing ethical leadership, we mentioned the reported benefits linked to this leadership style: pro-social behaviors [38]; psychological well-being and employees’ job satisfaction [39,40]; the creation of an ethical and familiar work climate that favors the participation of the workers [41]; organizational commitment, emotional commitment, normative commitment, organizational citizenship behavior, work performance, work commitment and organizational identification [42]; and a decrease in immoral behaviors in subordinates [43].

Similarly, some of the traits of the biomimetic leader are inspired by or extracted from behaviors associated with the styles of servant leadership (honesty, commitment, humility) and authentic leadership (transparency, loyalty, humility, honesty, etc.). When analyzing these leadership styles, we pointed out that some studies have shown the organizational effects of such leadership styles. Thus, the practice of servant leadership is positively associated with organizational commitment [48,49] and with the civic behavior of the followers [50,51]. Likewise, authentic leadership has been empirically related to the generation of trust, optimism, resilience and moral strength [31], and to a greater social identification with the group and the organizational principles [32]. We can expect the benefits associated with these leadership types to be present in organizations that promote biomimetic leadership.

Moreover, biomimetic leadership must be strongly linked to the sustainable development of the organizations that promote it. The concept of sustainable development refers to “one that meets the needs of the present without compromising the ability of future generations to meet their own needs” [129] (p. 23). The practical way in which companies have incorporated the concept of sustainability into their management systems is through the triple bottom line (TBL); this consists of extending traditional accountability, which shows the global net profitability, to a “triple result” account that includes the economic, social and environmental aspects of the organization [130,131]. Elkington [130] addressed the TBL with the aim of expanding the environmental agenda and integrating the economic and social aspects. Thus, profits, people and the planet are included, to measure the performance and success of an organization in a more consistent and coherent manner [132]. Consistency and coherence are inherent to the concept of sustainability proposed by Elkington, since the TBL is explicitly based on the integration of the three lines or dimensions, emphasizing all of them equally. However, balance is required, since the three pillars represent a necessary, although insufficient, condition for sustainable development [133]. Therefore, although the TBL perspective implies balancing the aspects of ecological, social and economic sustainability under the assumption that each of the three lines must be visible and healthy [134], the simultaneous integration of economic, social and environmental goals into a sustainable management strategy poses a managerial challenge to companies. We understand that those companies with biomimetic leaders will be better prepared to carry out strategic management that coherently coordinates and integrates the three dimensions of sustainability: economic, social and environmental. According to our characterization of biomimetic leaders, these leaders act from a holistic viewpoint, with the aim of attending to the needs of all the groups involved and integrating all perspectives.

Obviously, this work is not without limitations. From our point of view, one of the main attractive elements of this study, its radical novelty, can be considered as a limitation, since it is a new leadership model, and thus there are no theoretical scientific studies or business praxis to back our proposition. Therefore, it will be necessary for leaders of organizations to be previously and personally educated about the importance of being in favor of nature, without jeopardizing the corporate results. In this sense, it may be useful to present the biomimetic leadership model to leaders, in order to allow them to compare their current reality with the ideal profile, and then plan their journey toward strengthening their profile according to the new model of biomimetic leadership.

This investigation produced a seminal work in the field of leadership and organizational theory. Therefore, there is still much to do for the development of compact and thorough knowledge about biomimicry as applied to leadership. Future research lines derived from this study may include the development of a measurement scale that allows validating the characteristics of the biomimetic leader and empirically demonstrating the benefits of this leadership style for the organization.

## Figures and Tables

**Table 1:** Summary of the leadership models.

Leadership	Main Characteristics *	Published Studies
Transformational	**Altruism**, virtue, charisma, commitment to the common good, motivation, **creativity**, **individualized consideration**	Avolio and Locke (2002) [26] Bass (1985) [25] Bass and Steidlmeier (1999) [29] Van Dierendonck and Patterson (2015) [28]
Authentic	Self-awareness, **transparency in relationships**, moral perspective development, balanced processing, integrity, **honesty**, reliability, **humility**	Ilies, Morgeson and Nahrgang (2005) [32] Walumbwa et al. (2008) [30]
	Trust, optimism, **resilience**, moral strength, social identification with group and organizational principles Self-transcendence, **honesty**, **loyalty**, equality	Avolio and Gardner (2005) [31] Ilies, Morgeson and Nahrgang (2005) [32]
Ethical	Promotion of pro-social behaviors, increase of work satisfaction and psychological well-being. Defines what is fair and true **Honesty**, trust, listening	Avey, Wernsing and Palanski (2012) [40] Brown and Treviño (2006) [81] Brown, Treviño and Harrison (2005) [36] Kacmar et al. (2013) [38] Xu, Loi and Ngo (2016) [82]
Servant	**Honesty**, **commitment**, civic behavior, social communication and integration, co-responsibility, integrity, **humility**, reliability	Barbuto and Wheeler (2006) [54] Cerit (2010) [48] Chiniara and Bentein (2016) [50] Ehrhart (2004) [55] Greenleaf (1970) [44] Lapointe and Vandenberghe (2018) [49] Liden et al. (2008) [47] Ling, Lin and Wu (2016) [83] Van Dierendonck (2010) [45] Walumba, Hartnell and Oke (2010) [51]
Sustainable	“Bee” type vs. “lobster” type; psychological intelligence, psychological well-being, **ethical behavior**, responsibility for the environment, CSR, **innovation and creativity**, quality, passion, **personal and** **professional humility**, reflective capacity, introspection	Avery and Bergsteiner (2011) [59] Casserley and Critchley (2010) [84] Gerard, McMillan and D’Annunzio-Green (2017) [56] Kantabutra and Avery (2013) [58]
Creative	Novelty, openness to new realities, **creativity** as a personal decision. **Deferring judgment**, **keeping** **an open mind**, **thinking divergently**. Persistence, **curiosity**, interest for complexity, preference for autonomy and high energy	Basadur (2004) [63] DiLiello and Houghton (2006) [64] Palus and Hort (1996) [62] Sternberg, Kaufman and Pretz, (2003) [61]
Distributed	Shared leadership based on the activity and the profile of the members of the team **Holistic perspective**, concerted action, exchange, collaboration, dispersion, democratization, orienting, creating and maintaining motivation, managing changes **Moral authority**, persuasion, conciliation	DeMatthews (2014) [65] Longo (2008) [66] Spillane, Halverson and Diamond (2004) [85]
Spiritual	Integrity, **honesty**, **compassion**, **respect**, **humility**, transcendence, spirituality, **life sense**, far-sightedness	Benefiel (2005) [72] Blanch et al. (2016) [71] Fry (2003) [67] Morales-Sánchez and Cabello-Medina (2015) [69] Reave (2005) [68] Sharma (2003) [70]
Holistic	Distributed leadership, teamwork, goal orientation, quality control, **collaboration with external** **organizations** Participation, empowerment, **commitment**	Best (2011) [73] Korhonen et al. (2014) [74] Niemi (2015) [75]
Innovative	Persistence to solve problems, **combining different** **areas of knowledge**, **desiring change**, paying attention, personalization, image creation, fair play, collaborative inquiry, craftsmanship, resistance to ambiguities and paradoxes	Bornay-Barrachina (2013) [78] Ditkoff (2008) [76] González-Romá (2008) [77]
Regenerative	**Integration of different disciplines** **Transformative ethics** Presence, coherence, patience, generosity, silence	Hutchins and Storm (2019) [79] Ripper Kós et al. (2017) [86]

Source: developed by the authors from the literature review. * The traits that were considered for the characterization of the biomimetic leader are shown in bold type.

**Table 2:** Comparison between the traits of the biomimetic leader and the 10 key elements of a mature system.

Trait	Key Element of the Mature Ecosystem (Benyus) [2]	Relationship
Creativity	1. Uses waste as a resource 2. Diversifies and cooperates to fully use the habitat	Novelty, openness to new realities, creativity as a personal decision Deferring judgment, keeping an open mind, thinking divergently Persistence, curiosity, interest for complexity, preference for autonomy and high energy
		*Basadur (2004)* [63] *DiLiello and Houghton (2006)* [64] *Palus and Horth (1996)* [62] *Sternberg, Kaufman and Pretz (2003)* [61]
Ethics	3. Accumulates and uses energy efficiently 4. Optimizes, does not maximize 5. Uses materials with moderation 7. Does not deplete resources 10. Shops locally	Pro-social behaviors, defining what is fair
		*Avey, Wernsing and Palanski (2012)* [40] *Brown and Treviño (2006)* [81] *Brown, Treviño and Harrison (2005)* [36] *Kacmar et al. (2013)* [38] *Xu, Loi and Ngo (2016)* [82]
Flexibility	1. Uses waste as a resource 8. Maintains a balance with the biosphere 10. Shops locally	Responsibility for the environment
		*Avery and Bergsteiner (2011)* [59] *Casserley and Critchley (2010)* [84] *Kantabutra and Avery (2013)* [58] *Leroy, Palanski and Simons (2012)* [87] *Sabbaghi, Gerald and Hipskind S. J. (2013)* [88]
		Social identification with the group and organizational principles
		*Avolio and Gardner (2005)* [31] *Ilies, Morgeson and Nahrgang (2005)* [32]
		Exchange, collaboration, dispersion, democratization, orienting, creating and maintaining motivation, managing changes
		*DeMatthews (2014)* [65] *Longo (2008)* [66] *Spillane, Halverson and Diamond (2004)* [85]
Generosity	1. Uses waste as a resource 2. Diversifies and cooperates to fully use the habitat 5. Uses materials with moderation 7. Does not deplete resources	Commitment to the common good
		*Avolio, Bass and Jung (1999)* [89] *Barbuto and Wheeler (2006)* [54] *Bass and Steidlmeier (1999)* [29] *Hunter et al. (2013)*[90] *Melé (2012)* [35] *van Dierendonck and Patterson (2015)* [28]
Honesty	3. Accumulates and uses energy efficiently 4. Optimizes, does not maximize 7. Does not deplete the resources 8. Maintains a balance with the biosphere 9. Operates based on information 10. Shops locally	Honesty, commitment, civic behavior, co-responsibility, integrity, humility, reliability
		*Barbuto and Wheeler (2006)* [54] *Cerit (2010)* [48] *Chiniara and Bentein (2016)* [50] *Clopton (2011)* [53] *Ehrhart (2007)* [55] *Greenleaf (1970)* [44] *Lapointe and Vandenberghe (2018)* [49] *Liden et al. (2008)* [47] *Ling, Lin and Wu (2016)* [83] *Melé (2009)* [46] *Van Dierendonck (2010)* [45] *Walumbwa et al. (2008)* [30]
Humility	2. Diversifies and cooperates to fully use the habitat 6. Keeps his/her nest clean 7. Does not deplete resources 9. Operates based on information	Pro-social behaviors
		*Avey, Wernsing and Palanski (2012)* [40]
Loyalty/commitment	2. Diversifies and cooperates to fully use the habitat 3. Accumulates and uses energy efficiently 4. Optimizes, does not maximize 5. Uses materials with moderation 6. Keeps his/her nests clean 7. Does not deplete the resources 8. Maintains a balance with the biosphere 9. Operates based on information 10. Shops locally	Social communication and integration
		*Avey, Wernsing and Palanski (2012)* [40] *Barbuto and Wheeler (2006)* [54] *Cerit (2010)* [48] *Chiniara and Bentein (2016)* [50] *Clopton (2011)* [53] *Ehrhart (2007)* [55] *Greenleaf (1970)* [44] *Lapointe and Vandenberghe (2018)* [49] *Liden, Wayne, Zhao and Henderson (2008)* [47] *Ling, Liv and Wu (2016)* [52] *Melé (2009)* [46] *Van Dierendonck (2010)* [45]
Resilience	8. Maintains a balance with the biosphere	Trust, optimism, resilience, moral strength, social identification with the group and organizational principles
		*Avolio and Gardner (2005)* [31] *Ilies, Morgeson and Nahrgang (2005)* [32] *Reeves and Martin (2017)* [91]
Holistic view	1. Uses waste as a resource 2. Diversifies and cooperates to fully use the habitat 4. Optimizes, does not maximize 8. Maintains a balance with the biosphere	Distributed leadership, teamwork, goal orientation, quality control, collaboration with external organizations. Participation, empowerment, commitment
		*Best (2011)* [73] *Korhonen et al. (2014)* [74] *Niemi (2015)* [75]

Source: developed by authors.

**Table 3:** States of the self and personality traits.

State of the Self-Based on the Transactional Analysis	Definition	Associated Traits
Parent	*Internalized elements*. Truths recorded during childhood and internalized, “how things must be done”	Ethics Generosity Honesty Loyalty/Commitment
Adult	*Experienced elements*. Transformation of stimuli into elements of information, ordering and filing this information based on the acquired experience. Management of internal and external data for decision-making	Flexibility Humility Resilience Holistic view
Child	*Lived elements*. Feelings, emotions. Recalled memories that reproduce what the person lived, heard, felt and understood.	Creativity Sense of esthetics Sense of humor

Source: developed by the authors.

**Table 4:** Traits of the biomimetic leader.

	Trait	Definition
1	Sense of aesthetics	Enjoys observing nature; absorbs the beauty of nature with all his/her senses; makes new real or symbolic compositions based on elements he/she regards as beautiful. Appreciates the beauty of things, of daily life, or is interested in aspects of life such as nature, art, and science.
2	Creativity	Has the capacity to create and modify projects, products, services.
3	Ethics	Is aware of how his/her thoughts, words and actions affect his/her happiness and that of others. Operates based on principles and values of moral excellence. Feels and acts in coherence with moral values and good professional habits and practices, respecting the organizational policies. Feels and acts in this way at all times, in both their professional and personal life, even against the supposed interests of his/her own or of the sector/organization to which he/she belongs, since good habits and moral values are above his/her actions, and the company desires and understands this.
4	Flexibility	Adapts to changes or variations according to the circumstances or needs.
5	Generosity	Acts with magnanimity and nobility of mind. Offers part of the goods he/she possesses to others, including time and knowledge.
6	Honesty	Has uprightness of mind, integrity in his/her actions. Shows coherence between what he/she thinks, says and does. A straightforward person, coherent and truthful in his/her thoughts, words and actions.
7	Humility	Knows his/her own limitations and weaknesses and acts according to this knowledge; enjoys learning from others and from nature.
8	Loyalty/Commitment	Complies with the laws of fidelity and honor. Is willing to accept his/her own responsibilities and can respond to the demands of the job and to the duties required by the organization, the people that work in it and society in general, with a positive and spirited attitude.
9	Resilience	Can succeed under adverse conditions. Can tolerate failure.
10	Sense of humor	Can easily present, judge or comment on reality, highlighting the comic, positive or absurd side of things. Likes laughing and healthy joking, smiles frequently and always looks on the bright side of life.
11	Holistic view	Has a conception based on the total and global integration of a concept or situation; is versatile, open-minded and in constant growth.

Source: developed by the authors.

**Table 5:** Scores of the traits of the biomimetic leader, according to the expert panel.

	Trait	Score (Max 90)	Mean	Standard Deviation
1	Ethics	85	4.72	0.57
2	Holistic view	83	4.61	0.61
3	Generosity	80	4.44	0.86
4	Loyalty/Commitment	80	4.44	0.70
5	Honesty	79	4.39	1.24
6	Humility	79	4.39	0.85
7	Sense of esthetics	78	4.33	0.77
8	Creativity	77	4.28	0.75
9	Flexibility	77	4.28	0.89
10	Resilience	74	4.11	0.83
11	Sense of humor	69	3.83	1.10

Source: developed by the authors.

## Data Availability

Not applicable.

## Appendix A. Profiles of the Experts

- Marketing and Sales Director in a group of consulting services;
- Regional Director in an IT company/Dean of the Faculty of Economics, private university;
- Director of Innovation in a city council with more than 200,000 inhabitants;
- Dean of the Faculty of Economics, private university;
- Head of Reputation and Sustainability in a multinational energy company;
- Vice President and General Counsel in international health organization;
- Managing Director in a specialized consultancy firm focused on the work environment, commitment and culture;
- Director of Human Resources in an international health company in the optical sector and National President of a professional organization;
- Editor of an international journal of science;
- Director of Human Resources in a national energy company, member of the Board of Directors in two Professional Associations, and member of the Labor Court of an Autonomous Community;
- Professor of Ethics at a private university;
- Head of Human Resources and Corporate Social Responsibility in the most awarded SME in Spain in the tourism sector;
- Regional Director of a large national foundation (awarded best manager of the year in Spain);
- Executive President of a group of companies focused on professional services and business solutions;
- Director of Strategy and Corporate Social Responsibility at the Catalan Regional Institute of Health;
- Director of Organizational Development at a consulting firm in Israel with activities in various countries/Professor of Organizational Development at an American university;
- Director of Business Development in a large bank/a professor specializing in securities at a private university;
- Consultant in people management strategy and founding partner of the fourth sector/People and Talent Director in a national service company.
